# Integrative management of chronic kidney disease: A case study combining siddha and modern medicine

**DOI:** 10.6026/973206300214344

**Published:** 2025-12-15

**Authors:** Senthan Tamilselvana, Varshini Kumar, Mariappan Andic, Radha Sudalaimanid

**Affiliations:** 1 Department of Gunapadam, National Institute of Siddha, Affiliated to the Tamil Nadu Dr. MGR Medical University, Chennai-47, Tamil Nadu, India; 2 Department of Noi Naadal, National Institute of Siddha, Affiliated to the Tamil Nadu Dr. MGR Medical University, Chennai-47, Tamil Nadu, India; 3 Siddha Government Primary Health Center, Agastheeswaram, Tamil Nadu, India

**Keywords:** Chronic kidney disease, Siddha, integrative medicine, nephroprotective, renal fluid retention disorder (*Neeradaipu Noi*)

## Abstract

Chronic kidney disease (CKD) is a progressive disorder frequently associated with long standing Type 2 Diabetes Mellitus (DM) and Systemic Hypertension (SHTN). A 53-year-old male with an 8-year history of DM and a 5-year history of SHTN presented with pedal edema, frothy urination, dyspnoea and generalized itching, with no improvement from allopathic treatment and he refused dialysis. He opted for Siddha medicine, receiving Polyherbal nephroprotective decoction, polyherbal iron enriched decoction, calcined pearl-based formulation and calcined Herbo mineral formulation, while continuing Metformin and Amlodipine. After two and a half months, the patient showed significant clinical and biochemical improvements, including reduced serum creatinine, resolution of edema and relief from urinary and respiratory symptoms. Thus, we show the potential of Siddha medicine as a nephroprotective and integrative approach in managing CKD alongside allopathic treatment.

## Background:

According to the International Classification of Diseases, 11th Revision (ICD-11), the code GB61 designates chronic kidney disease (CKD). This classification is provided by the World Health Organization (WHO) and falls under the category of diseases affecting the genitourinary system [1]. CKD is defined by structural or functional abnormalities in the kidney or a persistent decrease in the Glomerular Filtration Rate (GFR) to less than 60 mL/min/1.73 m^2^ for at least three months [2, 3]. According to the Global Burden of Disease 2022 report, chronic kidney disease (CKD) has become the sixth leading cause of death worldwide among adults aged 19 to 64, with a mortality rate of 19.2 per 100,000 people. Its primary risk factors-hypertension and diabetes mellitus-are the first and third leading causes of death in this age group, respectively [4, 5]. The prevalence of CKD in the general population has enormously increased and in India, it is found to be 13.2 %. [6]. Less than half (47.9%) of the patients were prescribed renin-angiotensin-aldosterone system blockers. Among diabetic CKD patients, 25.7% were prescribed metformin. Statins were prescribed to only 40.4% of the cohort and among patients with anaemia, 31.1% received iron supplements, while 2.8% were prescribed erythropoiesis-stimulating agents [7]. Chronic kidney disease presents with fluid and electrolyte imbalances, cardiovascular issues, anemia, neuromuscular, gastrointestinal, nutritional, endocrine and metabolic disorders, affecting multiple systems and overall physiological function [8]. Dialysis and kidney transplantation are two therapeutic options for patients with chronic kidney disease (CKD). Studies revealed that patients undergoing haemodialysis were more likely to report leg muscle spasms, dry and itchy skin and blood pressure fluctuations compared to those undergoing peritoneal dialysis. Furthermore, haemodialysis patients experienced poorer sleep quality, more pain and greater challenges with sexual activity [9].

The majority of existing research consistently shows that patients undergoing haemodialysis experience a marked deterioration in their quality of life [10]. So, they needed conservative management to cut the complications. It was compensated by Siddha medicine. Chronic kidney disease (CKD) and Siddha are not directly related. Accordingly, it falls under renal fluid retention disorder (*Neeradaipu Noi*) based on clinical presentation and its symptoms are bagginess under eyes (*Kan rabbai veengi kanneer vadithal*), dyspnoea on exertion (*Maarbu nonthu moochu thadumaral*), Nausea (*Vanthi*), Anaemia (*Udal veluththal*), Puffiness of the face (*Muga veekam*), edema of the foot (*kaal vayiru miga oothuthal*). According to siddha principles, this condition corresponds with a disorder arising from dominant vatha imbalance associated with pitham, kabam reflecting a tridosha imbalance. Accordingly therapeutic management is based on assessment of humoral vitiation, disease stage and drug properties [11].

## Patient information:

A 53-year-old male patient working in a Xerox shop visited the National Institute of Siddha, Department of Gunapadam Outpatient Department, with the primary complaints of pitting edema in the bilateral dorsum of the foot, frothy urination, dyspnoea on exertion, nausea and itching all over the body for 6 months. He was a known case of Type 2 DM (Diabetes Mellitus) and SHTN (Systemic Hypertension) for 8 years and 5 years, respectively, for which he was under T. metformin 500 mg and Amlodipine 5mg. He had no relevant family history. He withdrew both medicines for 6 months without physician consultation. He was asymptomatic during this period. Gradually, he developed pitting edema in the bilateral dorsum of the foot, followed by frothy urination. Although a modern consultant found that his blood urea and creatinine were elevated, they prescribed allopathic medication for the above complaint. However, his blood urea and creatinine levels, along with his symptoms, have persisted after three months of follow-up with allopathic medication. Other symptoms, such as breathing difficulties during mild walking, progressively emerged during this time. Later, he developed body-wide itching and occasionally felt nauseous. On further investigation, he was diagnosed with CKD and was treated accordingly. He was advised to undergo dialysis, but the patient was unwilling. Therefore, he sought Siddha management for his current condition. Table 1 (see PDF) depicts the timeline of the symptoms and management.

## Diagnostic assessment:

The patient was diagnosed with CKD based on constantly increasing serum creatinine and urea levels, along with classical symptoms such as pitting edema in the bilateral dorsum of the foot, frothy urination (a sign of protein in the urine), shortness of breath (common in fluid retention or anaemia), itching (caused by a buildup of toxins in the blood) and nausea (potentially from metabolic imbalances). The symptoms gradually worsened over six months. The patient had a history of DM for 8 years, one of the leading causes of CKD, which plays a major role in Kidney damage and leads to Diabetic Nephropathy. He also had a history of HTN for 5 years. Uncontrolled high blood pressure also accelerates the progression of CKD by damaging the kidneys' delicate filters due to elevated pressure. His decision to stop taking medications for six months without consulting a doctor led to poor blood sugar control and fluctuating blood pressure, which likely sped up the progression of kidney dysfunction.

## Diagnostic reasoning:

Serum creatinine is frequently used to diagnose CKD, but it is a poor marker of glomerular filtration rate (GFR) in the early stages of the disease [12]. GFR is the most accurate way to assess kidney function and, therefore, is considered the gold standard for CKD diagnosis. The estimation of eGFR through established equations plays a key role in diagnosing kidney disorders, staging chronic kidney disease and evaluating therapeutic outcomes.

## Prognostic characteristics:

Initially elevated creatinine at 6.0-8.0 mg/dL, indicating significant kidney impairment, reduced to 1.7 mg/dL after treatment, nearing normal levels, suggesting recovery of kidney function. Reduced urea from 145 mg/dL to 60.7 mg/dL, further reflecting improved renal function. The patient's kidney function has significantly improved, as evidenced by reduced creatinine and urea levels, showing a good prognosis. Figure 1 (see PDF) depicts the before and after level of renal function tests.

## Therapeutic interventions:

Table 2 (see PDF) depicts therapeutic management.

## Follow up and outcomes:

After the first sitting, symptomatic improvement was observed. Bilateral pitting edema present in the dorsum of the foot is occasionally present, frothy urination is reduced, serum creatinine and urea were reduced to 5.0 mg/dl and 79mg/dl. Then after 2 months of treatment, itching all over the body and Nausea reduced, Dyspnoea on exertion occasionally present. Serum creatinine and urea were reduced to 1.7 mg/dl and 60 mg/dl. The significant reduction in creatinine and urea levels and symptomatic improvement show a good prognosis. The patient had no recurrence of symptoms during the Siddha drugless follow-up of 6 months. Images 1- 3 show the before and after treatment values.

## Intervention adherence and tolerability:

A drug compliance form was maintained to ensure intervention adherence.

## Adverse and unanticipated events:

No unexpected or harmful events were observed throughout the treatment period.

## Discussion:

This case highlights the efficacy of an integrated approach that combined Siddha medicine and allopathy treatment in managing CKD for a 53-year-old male patient with a known case of Type 2 DM and SHTN. He presented with advanced CKD symptoms such as pitting edema, frothy urination, dyspnoea on exertion, nausea and itching all over the body, which persisted despite allopathic treatment for CKD, including advice to undergo dialysis. The patient's symptoms and biochemical markers (serum creatinine and blood urea) showed no improvement. His decision to take conservative management through Siddha medicine after refusing dialysis provides a unique opportunity to evaluate the role of traditional medicine in CKD management. Early diagnosis of CKD is essential for preventing progression. This case illustrates the difficulty of symptom recognition in the early stages, as CKD often remains asymptomatic until GFR drops significantly. The patient's delayed presentation and withdrawal from prescribed diabetes and hypertension medications further complicated his management. The progression of CKD, even with allopathic treatment, stresses the challenges of managing the condition, particularly when comorbidities like diabetes worsen glomerular damage. The patient demonstrated marked improvement in clinical symptoms, along with a significant reduction in serum creatinine (from 8.0 mg/dl to 1.7 mg/dl) and blood urea levels (from 145 mg/dl to 60 mg/dl), indicating substantial recovery of renal function. This improvement is notably superior when compared with the study by Acharya *et al.* where creatinine levels decreased from 7.57 mg/dl to 5.72 mg/dl over the course of one month [13]. The gradual reduction in biochemical markers and symptomatic improvement suggests that Siddha interventions, such as Polyherbal nephroprotective decoction (*Neermulli Kudineer*), polyherbal iron enriched decoction (*Mandoorathi Kudineer*), calcined pearl-based formulation (*Muthu Parpam*) and calcined Herbo mineral formulation (*Jeevaloga Chendooram*), may have contributed to maintaining kidney function and reducing CKD progression. Polyherbal nephroprotective decoction showed its nephroprotective and antioxidant properties; this formulation reduces lipid peroxidation and oxidative stress induced by cisplatin, which are critical factors in CKD progression [14]. Treatment strategies for CKD anaemia include using erythropoiesis-stimulating agents (ESAs), iron therapy, ESA resistance and blood transfusion [15]. As per the literature, Ferric Oxide (Mandooram) and its preparations are vital in treating Anaemia (Pandu) cases. Ferric oxides such as ferric and ferrous fractions provide sufficient iron to the living matter, which is needed for normal erythropoiesis [16]. The ingredients of polyherbal iron enriched decoction possess heat and its quality allows the herbs and minerals to penetrate up to the cellular level of the tissue, thus helping in cleansing the microchannels and its iron-rich composition may have addressed CKD-related anaemia by improving erythropoiesis and iron metabolism. Calcined pearl-based formulation in the traditional Siddha text *Theran tharu* and has also been indicated in the Siddha formulary of India-Part-1, a compendium of Siddha formulations. The gem pearl (Muthu) has been indicated for a variety of acute and chronic diseases like otorrhoea, otitis media, asthma, diabetes, eye diseases, piles and urinary diseases and various neuromuscular diseases [17]. Calcined Herbo mineral formulation is indicated for Acute and Chronic nephritis conditions [18]. The ingredients in Calcined Herbo mineral formulation, mainly Eclipta prostrata and Phyllanthus niruri, have strong nephroprotective activity [19, 20]. The Siddha management approach, in this case, involved a combination of Decoction (Kudineer), calcined formulation (Parpam) and herbal powder (Chooranam) formulations aimed at balancing the patient's trihumoral vitiation and restoring renal function. The continuation of allopathic medications (Amlodipine and Metformin) alongside Siddha treatments suggests that the integrated approach may have enhanced therapeutic outcomes. This highlights the potential for traditional medicine to bridge gaps in conventional CKD management, particularly in patients resistant to allopathic medication or unwilling to undergo dialysis. The patient's adherence to Siddha medications and lifestyle modifications may have influenced the outcomes, emphasizing the importance of patient compliance in chronic disease management. The lack of standardized protocols for Siddha medicine in CKD care poses a challenge for widespread adoption and integration into mainstream healthcare. Collaborative efforts between traditional and modern medical systems can help develop evidence-based, integrative approaches to CKD care.

## Patient perspectives:

The patient reported on 30.08.2024 at the OPD National Institute of Siddha, that he is highly satisfied with the treatment, noting a significant reduction in symptoms. His quality of life improved.

## Informed consent:

The patient provided written informed consent for the use of their clinical information and images in this publication. They acknowledge that while direct identifiers will be excluded and confidentiality will be maintained to the greatest extent possible, complete anonymity cannot be assured.

## Conclusion:

The combination of Siddha medicine and allopathy demonstrated significant clinical and biochemical improvements in this patient with CKD. Thus, we show the importance of exploring integrative approaches to chronic disease management, while underscoring the need for further research into traditional medical systems, such as Siddha.

## Limitations:

The single-case design limits the generalizability of the findings. Larger, controlled studies are needed to validate the efficacy and safety of Siddha interventions in CKD management. Cystatin C could be assessed to gain a more comprehensive understanding of the condition. Extending the follow-up period may also be beneficial for a thorough evaluation of the prognosis. Research should focus on understanding the molecular mechanisms underlying the nephroprotective effects of these Siddha formulations.

## Source of funding:

None
